# The Book World of Medicine and Science

**Published:** 1902-02-15

**Authors:** 


### THE BOOK WORLD OF MEDICINE AND SCIENCE.

THE COMPOSITION OF DUTCH BUTTER. By Dr. J. J. VAN RYN, Director to the Royal Agricultural Experimental Station at Maastricht. (London: Baillière, Tindall & Cox. 1902.)

HOLLAND is a large exporter of both butter and margarine to the United Kingdom, and it is therefore of great importance to English consumers to be assured that the former is a pure product manufactured from milk only. But, owing to the great difficulties in determining the amount of butter fats which ought to be found in pure butter, Dr. Van Ryn says "that Dutch butter, the origin of which placed its genuineness and purity beyond doubt, has been wrongly declared by foreign chemists to be a mixture of butter and margarine." Butter varies in composition very greatly at different times of the year, particularly in the autumn, and for this reason the Dutch Government ordered a full examination to be made of the Dutch butter produced in the latter months of the year, chiefly in order "to show English chemists the real position of affairs." The usual method of distinguishing butter from other animal fats is by determining the percentage of volatile and non-volatile fatty acids present, and if the former falls below a certain figure, the butter is presumed to be adulterated. Dr. Van Ryn has proved that undoubtedly pure butter in many cases does not contain this percentage of volatile fatty acids, and has carried his investigations further to determine the chief causes of this variation of composition. It appears that in October the percentage of volatile fatty acids is lowest, and then it rises steadily in November and December. Of 428 samples analysed during September and October, no less than 50 per cent. showed a saturation point of volatile fatty acids below the lowest limit generally accepted as a test of purity. On the other hand, of 166 samples analysed during the latter part of October and November, not one failed to satisfy this test. Dr. Van Ryn does not attach much importance to the period of lactation, as an influence affecting the composition of butter, but attributes the variation in these months to the "stabling" and "feeding" of the cows, and thinks that the "abnormal chemical composition of Dutch butter during the autumn must be attributed to the circumstance that the farmers leave their cattle in the meadows until late in the year." The whole report is a most valuable contribution to the literature on this subject, but before a definite opinion can be given, it will be necessary to institute a similar series of analytical experiments both on home-made butter and also that imported from other countries during the same season of the year.

REPORT ON THE TREATMENT OF PLAGUE WITH PROFESSOR LUSTIG'S SERUM AT THE ARTHUR ROAD HOSPITAL. By KHAN BAHADUR, Dr. N. H. CHOKSY, Special Assistant Health Officer, Bombay Municipality. (Bombay: Printed at the *Times of India* Press. 1901. Pp. 37, with Charts of Plague Cases.)

THE report begins with a brief but interesting *résumé* of recent researches in bacterio-therapy in which is pointed out the reason why the serum treatment has not been as satisfactory in cholera, typhoid, and plague as in diphtheria. The latter disease is an intoxication, the bacteria remaining *in situ* at the point of inoculation and the symptoms being almost exclusively due to the action of a toxine excreted by the microbe as a sort of metabolic product, whereas in the former diseases the specific poisons of the bacilli are not excreted by them, but are retained in the protoplasm forming their substances. The epidemic of 1900-1901 showed an increasing virulence characterised by rapid extension of the local infection with multiple contiguous buboes, intense and rapid septicæmia, prolonged duration, tardy convalescence, and greater resistance to the action of serum. Pneumonic plague and plague without apparent buboes both showed a case mortality of 100 per cent., while multiple buboes ranked next with 79·53 per cent., single axillary buboes next with 76·71 per cent., and single buboes in the axilla were least fatal with 50 per cent. The septicæmic cases resisted the action of serum altogether, 55 dying and only two recovering, but in the non-septicæmic cases so treated 43 terminated fatally in a total of 76. The total number of cases treated by serum show a mortality rate of 67·30 per cent. as opposed to one of 77·16 per cent. in the non-serum cases. These results, although not so encouraging as anticipated, are nevertheless not to be despised, when we realise how high is the normal mortality from the disease.

THE IMPERIAL HEALTH MANUAL: BEING THE AUTHORISED ENGLISH TRANSLATION OF THE OFFICIAL HEALTH MANUAL ISSUED BY THE IMPERIAL HEALTH DEPARTMENT OF GERMANY. Edited by ANTHONY ROCHE, M.R.C.P.I., etc. London: (Baillière, Tindall and Cox. 1902. Price 3s. net.)

WE doubt if there is in the English language any other small popular handbook exactly covering the field occupied by the interesting little manual. We have little ambulance books by the dozen and popular physiology books by the score; but this is neither the one nor the other—or, rather, it is both, and much besides. It takes in everything which makes for or against health; it treats of all kinds of departures from health; it describes various methods of treatment and nursing and bandaging; it gives details about the rearing of children, about stoves and the construction of houses; about commerce and quarantine; about physical geography, geology, mustard plasters, and many other things; and, although it is undoubtedly an *olla podrida*, it is all very good.

With such a variety of subjects, and with only 300 small pages into which to crowd all his ideas, one need hardly say that the author has had to study the art of condensation; and although it must be confessed that all is very well done, an irritating sense of incompleteness is left after reading almost any portion of it. Its proper place would seem to be that of a text-book to be read by those who are receiving practical *vivâ voce* instruction on the subjects dealt with. It is carefully written and well translated, and is full of information, and if it produces in the reader a desire for greater detail, that is, perhaps, what it is meant to do.

